# Formation, Evolution, and Antioxidant Activity of Melanoidins in Black Garlic under Different Storage Conditions

**DOI:** 10.3390/foods12203727

**Published:** 2023-10-11

**Authors:** Bobo Wang, Yu Zhong, Danfeng Wang, Fanbing Meng, Yuncheng Li, Yun Deng

**Affiliations:** 1 Department of Food Science & Technology, Shanghai Jiao Tong University, 800 Dongchuan Road, Shanghai 200240, China; a1102266106@163.com (B.W.); zhongyu@sjtu.edu.cn (Y.Z.); llz26@sjtu.edu.cn (D.W.); 2 Shanghai Jiao Tong University Sichuan Research Institute, Chengdu 610213, China; 3 College of Food and Biological Engineering, Chengdu University, Chengdu 610106, China; mfb1020@163.com (F.M.); liyunchengs@126.com (Y.L.)

**Keywords:** melanoidin, black garlic, antioxidants, food-quality improvement, botanicals

## Abstract

Melanoidins (MLDs) are formed through the reaction of carbonyl compounds and amino compounds in the Maillard reaction (MR) during the heating or storage of food. In this study, the formation, chemical composition, and structural characteristics of black garlic (BG) MLDs stored at different temperatures (4 °C, 20 °C, and 35 °C) over a period of 6 months were investigated. The initial products of the MR formed more often at 4 °C and 20 °C, while higher temperatures (35 °C) promoted the reaction in the middle and late stages of the MR. The higher temperature promoted an increase in molecular weight and MLD content, which can be attributed to the increase in protein and phenolic content. Elemental analysis confirmed an increase in nitrogen (N) content and the continuous incorporation of nitrogen-rich substances into the skeleton. Amino acids, particularly aspartic acid and threonine, were the primary N-containing compounds involved in MLD formation. Additionally, the infrared analysis revealed that the changes in MLDs during storage were characterized by amide I and amide II groups. The MR enhanced the yields of heterocyclic compounds (from 56.60% to 78.89%), especially that of O-heterocyclic compounds, at the higher temperature according to Py-GC-MS analysis. Furthermore, the higher temperature enhanced the molecular weight, maximum height, and roughness of MLDs compared to the control. The antioxidant ability of MLDs was positively correlated with storage temperatures. In summary, temperature had an impact on the formation, evolution, and antioxidant activity of MLDs.

## 1. Introduction

Black garlic (BG) is a processed product of fresh garlic. It has gained attention from customers due to its abundance of bioactive substances [1], such as melanoidins (MLDs) and vitamins, and potential function in the treatment of and reduction in various disease risks, including hypertension [2], cancer [3], inflammation [4], and obesity [1]. Previous research has proved that the changes in color, flavor, and compositions can mainly be attributed to the Maillard reaction (MR) [5], which occurs during the heating and storage of foods [6]. The MR involves a series of interactions between the carbonyl group in a reducing sugar or oxidized lipid and the amino group in amino acids, peptides, or proteins. It was reported that the cysteine content in BG is 5 times higher than that in white garlic [7]. However, the changes in amino acids in BG MLDs during storage are little-studied.

MLDs have complex compositions and structures due to different reactants and diverse reactions. A few studies have shown that the skeleton of MLDs is established through the cross-linking of carbohydrate degradation products with proteins and other macromolecular substances [8,9]. MLD structural changes due to changes in noncovalent interactions were confirmed via FT-IR [10]. Pyrolysis–gas chromatography–mass spectrometry (Py-GC-MS) was used to infer the compositions, structures, and chemical processes of the samples from the pyrolysis products and thus to infer the reaction mechanism [11]. The compositions of BG MLDs were analyzed and found to contain furans, aldehydes, alcohols, ketones, hetero -cycles, and aromatics [12]. However, the compositional changes in BG during MLD storage are still unknown.

The structures of MLDs are affected by a series of factors, especially temperature and time [13]. The activation energy of the reaction increases with increasing temperature. Previous studies have demonstrated that the content of reactive substrate in the system is enhanced with an increase in reaction temperature and the MR speed is accelerated [14]. Compared with low-temperature fermentation, high-temperature treatment results in a higher MLD content and molecular weight [15]. Additionally, reaction time is another important factor affecting the MR; extending the reaction time has been found to enhance the abundance and types of MLDs [16] and also facilitates the incorporation of phenol into the skeleton of MLDs [17]. Although many studies have proved that the structures and antioxidant ability of MLDs change according to the temperature, there are few studies on the specific changes occurring during the storage of BG. Furthermore, it has been indicated that MLDs present different biological activities, especially in terms of antioxidant capacity [18]. The MLDs of BG have strong antioxidant properties [10], but the antioxidant capacity of BG MLD storage has not been clarified yet.

The experiment was a single-factor experiment (temperature). Three temperatures were set, and six time points were taken at each temperature for the experiment. Our studies confirmed that the alterations in the compositions and structures of MLDs during storage. Then, the changes in antioxidant activity were evaluated via DPPH, ABTS, and a reducing power assay. The present study lays the foundation for future research on the formation mechanism and biological activities of MLDs.

## 2. Materials and Methods

### 2.1. Materials

BG was produced, based on the enterprise standard (QDPH 0001S-2019), by Dali Pinhong Plateau Agricultural Science and Technology Development (Yunnan Province, Dali, China). All chemicals were of analytical grade and purchased from Sigma Chemical Co. (St. Louis, MO, USA). BG was stored at 4 °C, 20 °C, and 35 °C for 6 months in PETB bottles.

### 2.2. Sample Preparation

MLD sample preparation was carried out based on the previous method of Zhao et al. (2011) [10] with some modifications. Peeled black garlic was mixed with distilled water at 1:9 (*w*/*w*). Then, the mixture was ground for 4 min and left to rest in an ultrasonic disintegrator (s10H, zealway, Xiamen, China) for 20 min. The supernatant was degreased twice with dichloromethane at 3:2 (*w*/*w*). The solution (10 mL) was dialyzed with an ultrafiltration membrane (MLD44-12, Viskase, Lombard, IL, USA) of 3 kDa in distilled water (1 L). The water was replaced three times during dialysis. MLDs were freeze-dried (FD-250101 GT, FTFDS, Hangzhou, China) and preserved at −80 °C in a dry environment until analysis.

### 2.3. Absorbance Measurement 

MLDs were measured as in the previous method [19] using a spectrophotometer (Epoch, BioTech Instruments, Winooski, VT, USA). MLDs (0.1 g) were dissolved in distilled water (10 mL), and the solution was mixed for 20 s. The absorption of MLDs was determined at 280, 325, and 420 nm against a blank containing only distilled water. 

### 2.4. Reaction Kinetics

The change in the absorbance of MLDs during storage can be simulated through first-order reactions.

(1) $$\ln A=\ln A_{0}-kt$$

where t is the storage time (month), A_0_ and A are the absorbance values at time zero and time t, and k is the first-order rate constant (month^−1^) under the corresponding storage conditions. 

### 2.5. Chemical Compositions

#### 2.5.1. Reducing Sugar

Sample solution (0.5 mg/mL) measuring 0.5 mL was added 1.0 mL of DNS. The sample was heated in a water bath at 100 °C for 5 min, and then water was added to reach a volume of 5 mL after cooling in the ice bath. Finally, the absorbance was measured at 540 nm.

#### 2.5.2. Protein

The Coomassie brilliant blue method was used to measure the content of protein in MLDs. The MLD solution (0.5 mg/mL, 1 mL) and the Coomassie brilliant blue reagents were mixed and left to rest for 10 min. The absorbance was measured at 595 nm.

#### 2.5.3. Total Phenolic (TPC)

The total phenolic (TPC) content in MLDs was determined using the Folin–Ciocalteu method as described by Babbar et al. (2011) [20]. About 200 μL of Folin–Ciocalteu reagent and the same amount of MLD solution were mixed for 5 min, and sodium carbonate (600 μL, 0.1 mol/L) was added at 75 °C for 30 min. The absorbance of sample was measured at 765 nm.

### 2.6. Elemental Analysis

Elemental analysis was performed on the element analyzer (Elementar vario EL Cube, Elememtar, Langenselbold, Germany). MLDs (3 mg) were wrapped with tin foil and completely burned at a temperature above 1150 °C. Then, the element signal was measured with a thermal conductivity detector to calculate the content of the samples. 

### 2.7. Py-GC-MS 

Pyrolysis–gas chromatography–mass analysis was carried out in a gas chromatograph equipped with a mass spectrometer (Model 5975C, Agilent, Santa Clara, CA, USA), as previously described by Rochat et al. (2007) [14]. The GC injection temperature was 300 °C. The GC heating program was as follows: The temperature was set at 60 °C for 3 min and then increased up to 300 °C for 10 min. The MLD sample (0.5 mg) was pyrolyzed at 550 °C. Pyrolysis products were determined on the basis of the National Institute of Standards and Technology (NIST) library (https://cheMLData.Nist.gov/ (accessed on 5 May 2023)), and pertinent literature was used to compare results. 

### 2.8. Molecular Weight Measurement 

The molecular weight of MLDs was determined via high-performance gel permeation chromatography (Waters 2695, Agilent, Santa Clara, CA, USA) with a UV detector and an automatic sample injector. All separations were carried out using chromatographic columns (TSKGgel G3000SWXL 7.8 × 300). Four standards were used for molecular weight calibration: Thyroglobulin bovine (670 kDa), γ-globulins from bovine blood (150 kDa), albumin chicken egg grade VI (44.3 kDa), and ribonuclease A type I-A (13.7 kDa). 

### 2.9. Fourier Transform Infrared Spectroscopy (FT-IR) Analysis 

FT-IR analysis was conducted with an FT-IR attenuated total reflection (ATR) spectrometer (Thermo Scientific, Waltham, MA, USA). Spectra records were kept at a resolution between 400 and 4000 cm^−1^ for 64 scans [21]. 

### 2.10. Morphology Analysis

The morphology of MLDs was determined via AFM (MFP 3D, Asylum Research, Oxford Instruments, Oxford, UK). The 50 μL diluted sample (25 μg/mL) was deposited onto a mica slide. Nitrogen was blown over the samples at room temperature for 1 min before analysis. 

### 2.11. Antioxidant Activity

The antioxidant activity was measured according to modified methods of Babbar et al. (2011) [20], including the DPPH, ABTS, and total reducing power tests. For the DPPH test, MLD solution (1 mL, 0.01 mg/mL) was mixed with DPPH solution (19 mL). The absorbance was measured at 515 nm after 20 min in the dark. For the ABTS assay, MLD solution (1 mL, 0.01 mg/mL) was mixed with ABTS solution (19 mL). The absorbance was measured at 734 nm after 20 min in the dark. For total reducing power, MLD solution (0.5 MLX, 1 mg/mL) was mixed with phosphate buffer (1.25 mL, 0.2 mol/L, Ph = 6.6) and 1.25 mL K_3_[Fe (CN)_6_] solution (1%, *w*/*v*) and then heated in a water bath at 50 °C for 20 min. The Cl_3_CCOOH solution (2.5 mL, 10%, *w*/*v*) was added and centrifuged at 4500 r/min for 10 min. The supernatant (2.5 mL) was mixed with 2.5 mL of deionized water and 0.5 mL of FeCl_3_ (0.1%, *w*/*v*). The absorbance at 700 nm was measured after mixing at room temperature for 10 min.

(2) $$DPPH\;radical\;scavenging\;activity\;(\%)=\left[1-\frac{A_{S}}{A_{0}}\right]\times 100\%$$

(3) $$ABTS\;radical\;scavenging\;activity\;(\%)=\left[1-\frac{A_{S}}{A_{0}}\right]\times 100\%$$

where A_s_ is the MLD absorbance and A_0_ is the absorbance of the control group.

### 2.12. Statistical Analysis 

All experiments were performed in triplicate. Statistical analysis was performed using the SPSS 21.0 software (IBM, Chicago, IL, USA). Statistical data were expressed as mean ± standard deviation. Significant differences among the means were determined via ANOVA corrected for multiple comparisons with Duncan’s multiple range test, and values of *p* < 0.05 were considered to show significant differences. 

## 3. Results and Discussions 

### 3.1. Absorbance Measurement 

**Figure 1:**
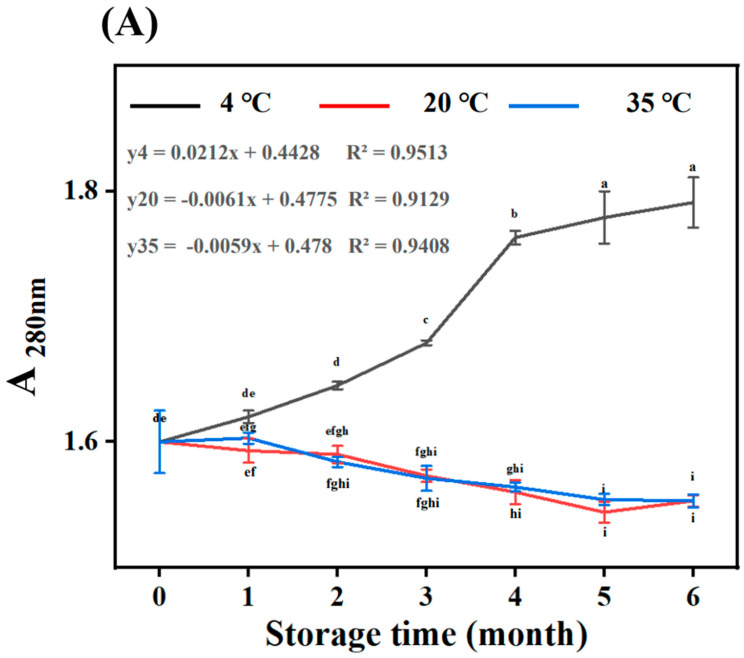

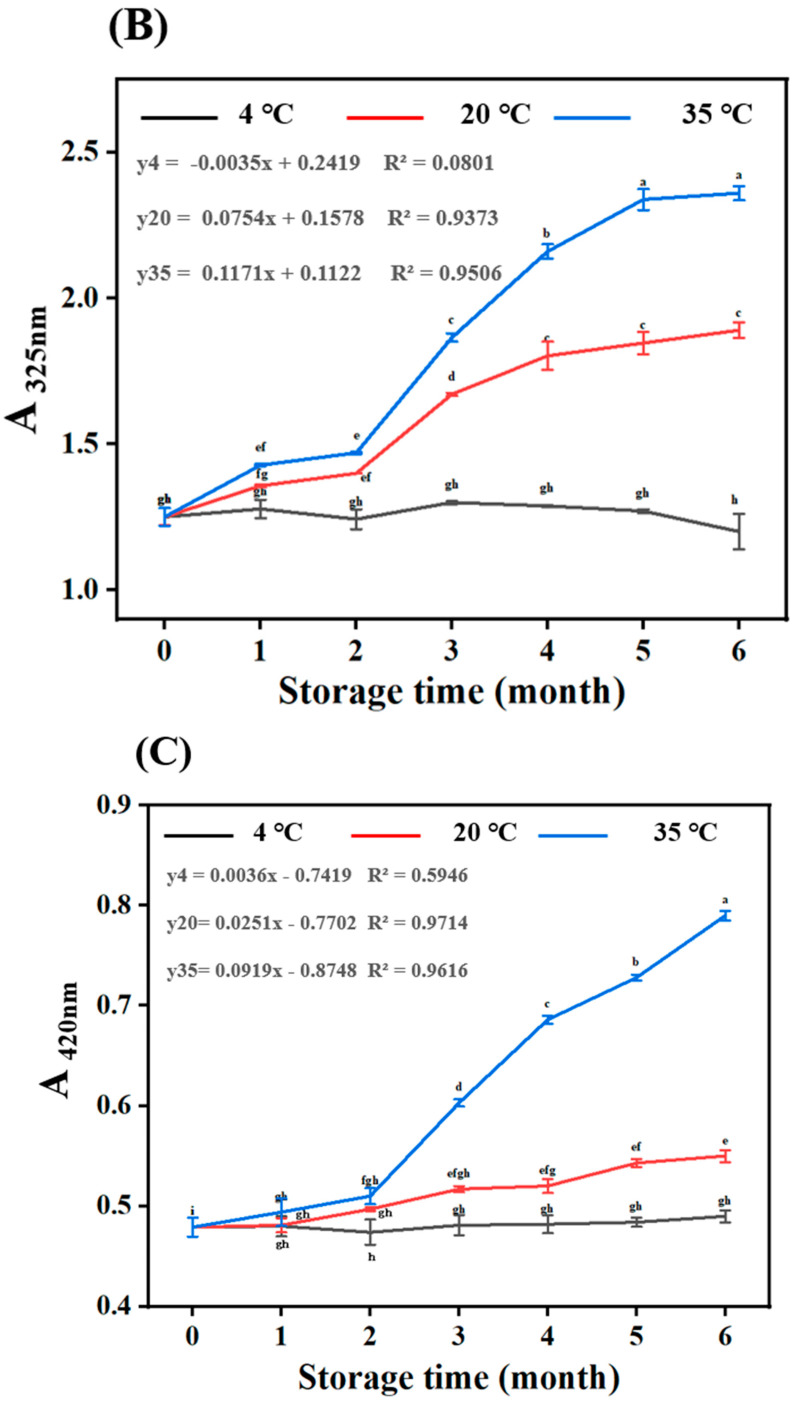
Melanoidin content evaluation and first-order reaction model at different temperatures at (**A**) 280, (**B**) 325, and (**C**) 420 nm. Different letters (a–i) within the same absorbance are significantly different at *p* < 0.05, as analyzed using Duncan’s multiple range test.

MLDs are the final product of the MR and appear as brown in aqueous solution. MLDs have a wide UV absorption range, covering almost the entire UV spectrum. In the present study, MLDs in BG were characterized via spectrophotometry to evaluate their properties. The absorbance of MLDs in BG under different storage conditions at 280 nm (A_280 nm_), 325 nm (A_325_ nm), and 420 nm (A_420_ nm) is shown in Figure 1. The increase in A_280_ nm at 4 °C was the most significant with the increase in temperature, while A_325_ nm and A_420_ nm increased more at 20 °C and 35 °C. After the same amount of time in storage, A_280_ nm decreased with the rising temperature, while A_325_ nm and A_420_ nm increased. The absorbance at 280 nm, 325 nm, and 420 nm corresponded to the initial, intermediate, and final stages of the MR, respectively. A_280_ nm also demonstrated that the colorless intermediate was produced via the condensation of glucosamine and Amadori rearrangement in the initial stage of the MR [22], and the furan structure and the heterocyclic compounds were incorporated into the MLD skeleton. 

Our results confirm that a large number of initial MR products accumulated during storage at 4 °C. Colorless substances in the intermediate stage (325 nm) were produced due to a variety of reactions, including amino acid degradation (Strecker degradation), sugar dehydration, and sugar fragmentation. Highly colored products were formed through aldol condensation, aldehyde amine condensation, and the formation of heterocyclic nitro compounds in the final stage (420 nm). The rate constant of the first-order reaction equation showed that the reaction rate was the fastest at 4 °C, which means the rate of MR product formation in the initial stage was fastest at 4 °C. The reaction rate in the middle and late stages of the MR increased with the increase in temperature. The reaction rate of MLDs stored at 35 °C was 1.55 times faster than the rate of those stored at 20 °C in the middle stage of the MR, while it was 3.66 times as fast as the rate of those stored at 20 °C in the later stage of the MR. Therefore, more initial-stage products formed at lower temperatures, while more MLDs were formed at the higher temperature over the same amount of storage time. Overall, the temperature effectively promoted the MR, thereby accelerating the generation of high-molecular-weight (HMW) MLDs. The results are similar to those found in a previous report by Yang et al. (2023), which showed that the content of MLDs in distillers’ grains increased with an increase in heating temperature [15]. They found that temperature promoted the production of MLDs more than time, which is similar to our results. For a better understanding of the effects caused by temperature on the MR, the specific change mechanism induced by temperature and the reaction mechanism in the final stage of the MR need to be investigated.

### 3.2. Chemical Compositions of MLDs under Storage at Different Temperatures

#### 3.2.1. Reducing Sugar

**Figure 2:**
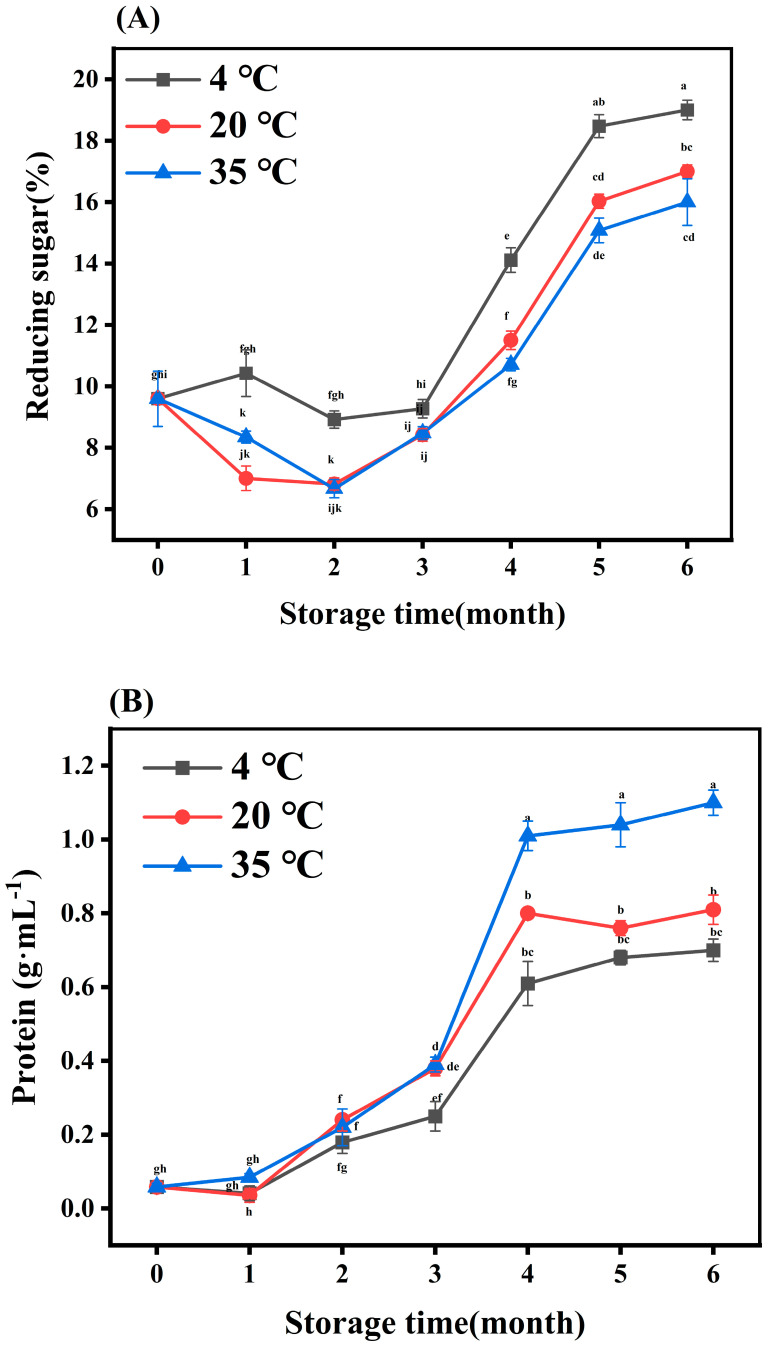

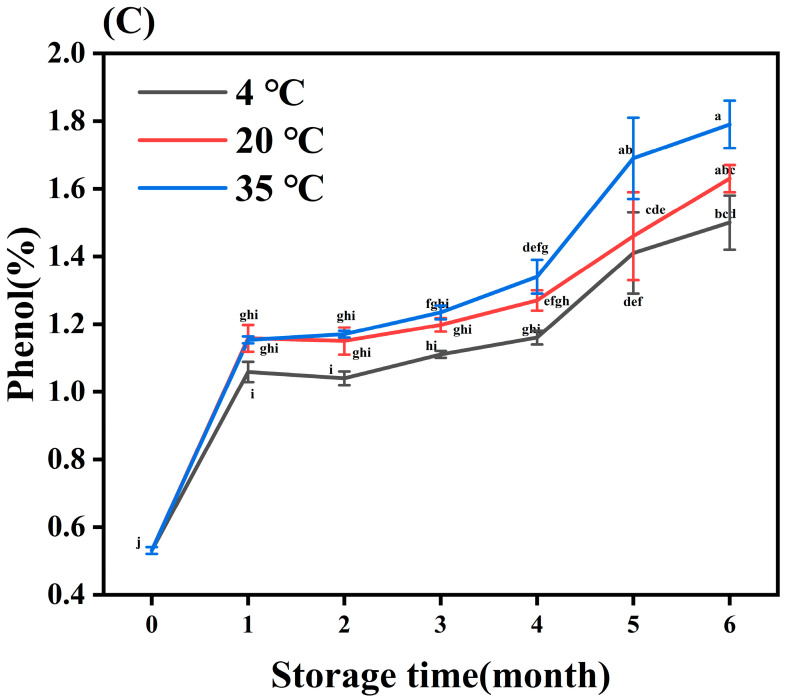
Chemical composition analysis of melanoidins stored at different temperatures for various durations. Content of (**A**) reducing sugar, (**B**) protein, and (**C**) phenol in melanoidins stored at 4 °C, 20 °C, and 35 °C. Different letters (a–k) within the same absorbance are significantly different at *p* < 0.05, as analyzed using Duncan’s multiple range test.

The specific chemical compositions of MLDs are unclear, but sugar and protein are considered to form the skeleton of MLDs. As shown in Figure 2A, the reducing sugar content increased during storage at the same temperature, which was the result of the ongoing MR and the incorporation of carbohydrates into the MLD structure. Interestingly, the reducing sugar proportion decreased with the elevation in storage temperature after the same amount of storage time, as a result of greater incorporation of the other components in the MLD structure. 

#### 3.2.2. Protein and Amino Acids

It could be seen that proteins were continuously added to the MLD structure as the MR progressed (as shown in Figure 2B), which is similar to Bekedam’s finding that the number of amino acids/proteins in dark coffee increased upon roasting [23]. The protein content was elevated with increasing temperature in the same storage period. However, the specific mechanism of protein change has not been clearly elucidated due to the complex reactions of protein during the fermentation of BG.

Free amino acids are precursor compounds formed by MLDs. They are mainly involved in Strecker degradation, and their consumption can indicate the extent of the MR [24]. The main amino acids produced under different storage conditions are shown in the Appendix A, Table A1. The free amino acid compositions at different storage times were roughly the same, while the content of these amino acids decreased during storage, especially that of aspartic acid and threonine. It was inferred that the rate of protein hydrolysis to amino acids was different, because protein degradation produced a large number of free amino acids involved in MLDs; first, they were fragmented into small molecules, and then they were finally polymerized into HMW MLDs. Additionally, this might be attributed to the extent different amino acids participated in the MR as a result of their structures and amino numbers. 

#### 3.2.3. Total Phenolic

Phenols are often bound to the MLD skeleton by covalent or noncovalent bonds [25]. The total phenolic content gradually increased with the rise in storage time and temperature, as shown in Figure 2C. Heat treatment increased the entry of phenols into the MLD skeleton. That was because macromolecular phenolic compounds decomposed into small molecules, releasing more phenolic hydroxyl, which could then participate in the MR. In their report, Quiroz-Reyes et al. (2018) observed that higher temperatures promoted the binding of phenolic compounds in the MLD structure, and this was also the main pathway for the degradation of cocoa bean phenols [26]. In summary, higher temperatures (35 °C) promoted an increase in carbohydrate, protein, and phenolic content, which was due to the increasing content of MLDs. 

### 3.3. Elemental Analysis 

A quantitative elemental analysis was used to estimate and compare the elemental compositions of MLDs (as shown in Table 1). At the same temperature, the content of O decreased, and the content of C, H, and N increased with the increase in time. At the same time, the contents of C, H, and N increased significantly with the increase in temperature. 

In summary, we identified that the increase in temperature and time caused the continuous construction of the MLD skeleton, which was caused by the continuous incorporation of carbon-rich and nitrogen-rich substances. The increasing integration of carbon-rich substances, such as reducing sugar and total phenol, led to the final polymeric MLD skeleton. The content of N increased during storage at high temperatures with a significant difference (*p* < 0.05). This result is in line with the observation made by Kang, who reported that N content increased during MLD formation from BG processing [27]. Compared with our previous studies, it was inferred that the nitrogen-rich substances that formed MLDs in black garlic might mainly come from protein, aspartic acid, and threonine. The decrease in O showed that the elimination of water resulted in the formation of double-bond and conjugated double-bond systems. Therefore, the larger MLD skeleton and increase in polymers affected their elemental compositions. 

### 3.4. Py-GC-MS Analysis

#### 3.4.1. Characteristics of Volatile Compounds Formed through MLD Pyrolysis 

The pyrolysis products of MLDs were identified via gas chromatography–mass spectrometry. The similarity between 132 components and the NIST library was more than 80%. The main components of the MLDs in BG can be divided into six categories, including heterocyclic compounds, acids, aliphatic hydrocarbons, ketones, alcohols, and aromatic compounds (as shown in the Appendix B, Table A2). Heterocyclic compounds were the main pyrolysis products, including N-heterocyclic compounds (pyrazoles and pyridines) and O-heterocyclic compounds (furan derivatives), which is consistent with the results of previous research [12]. The content of heterocyclic compounds was enhanced with increasing storage temperature. The content was the highest (78.89%) after 6 months of storage at 35 °C, being 39.38% higher than that of the untreated group. The content increased by 28.09% after storage at 20 °C, while the content changed less after storage at 4 °C. 

It was concluded that temperature promoted the production of heterocyclic compounds in the MR middle stage, during which the content of O-heterocyclic compounds was higher than that of N-heterocyclic compounds. Heterocyclic compounds were primarily produced through non-enzymatic browning, the MR, or pyrolysis of sulfide compounds. Additionally, the existence of small molecular substances (acids, alcohols) proved that there was a ring-opening fracture of the furan ring [28]. In summary, higher temperatures promoted the production of products in the middle and late stages of the MR.

#### 3.4.2. Principal Component Analysis (PCA), Partial Least Squares–Discriminant Analysis (PLS-DA) and Heat Map Analysis 

**Figure 3:**
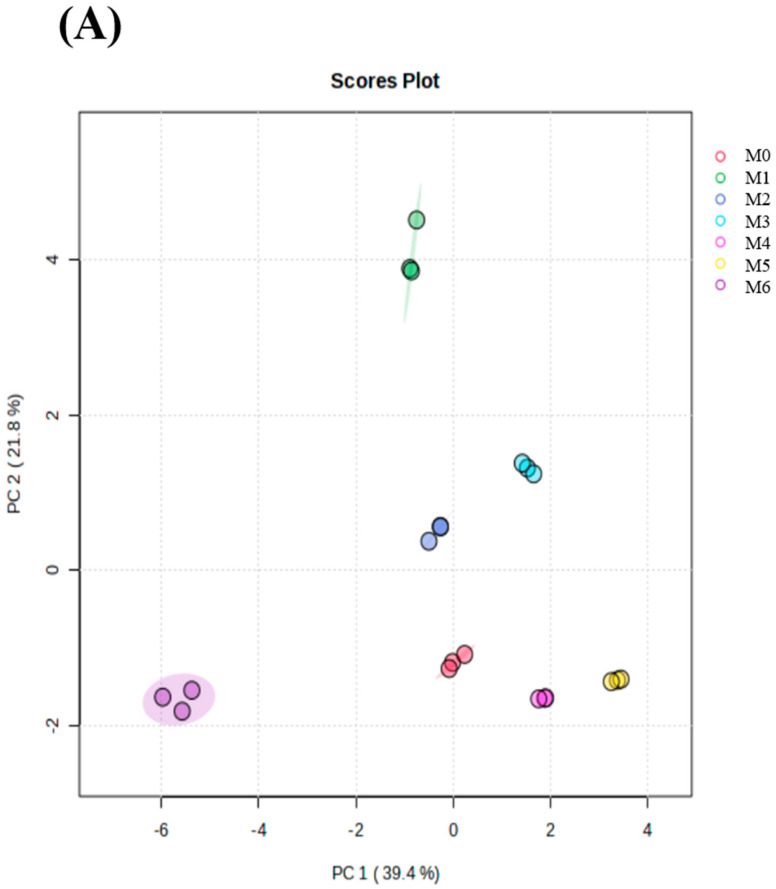

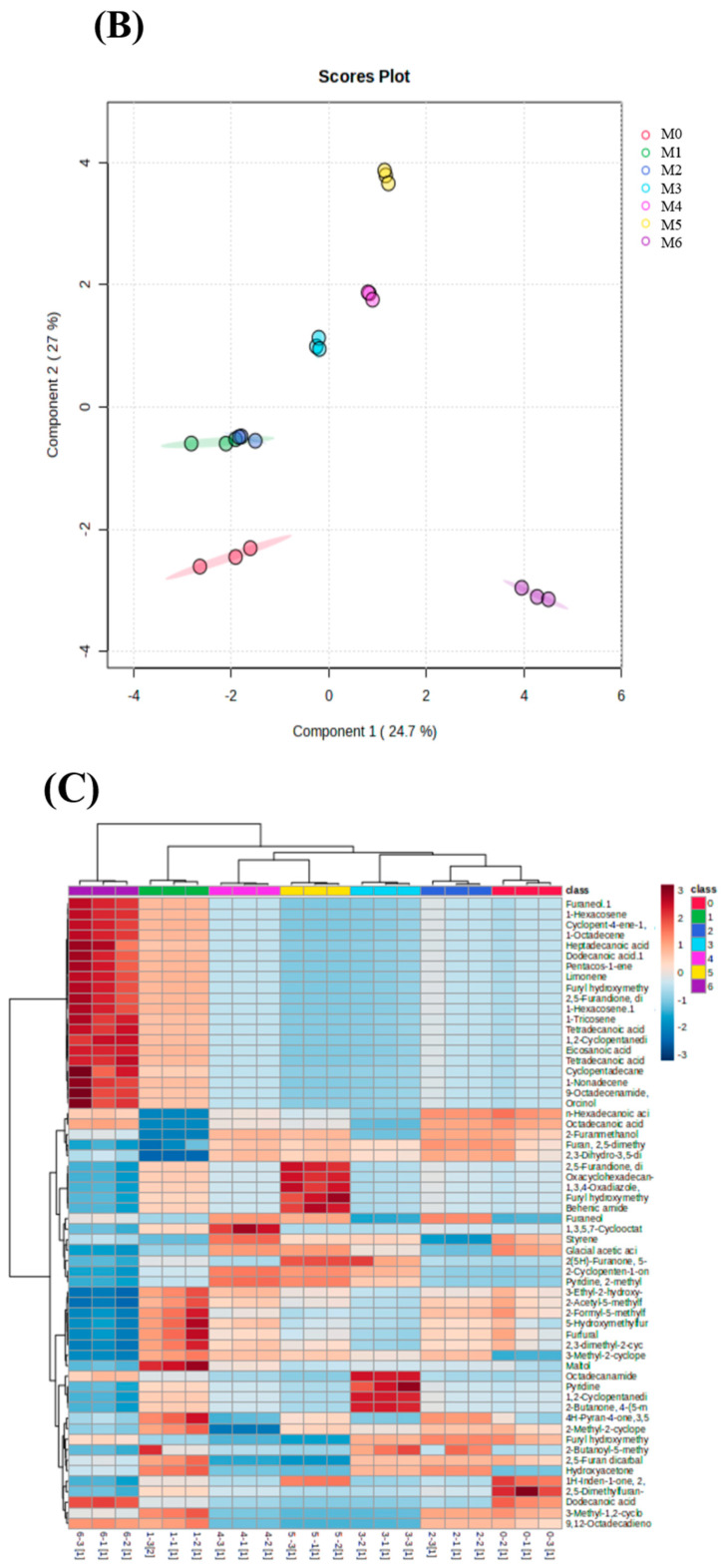

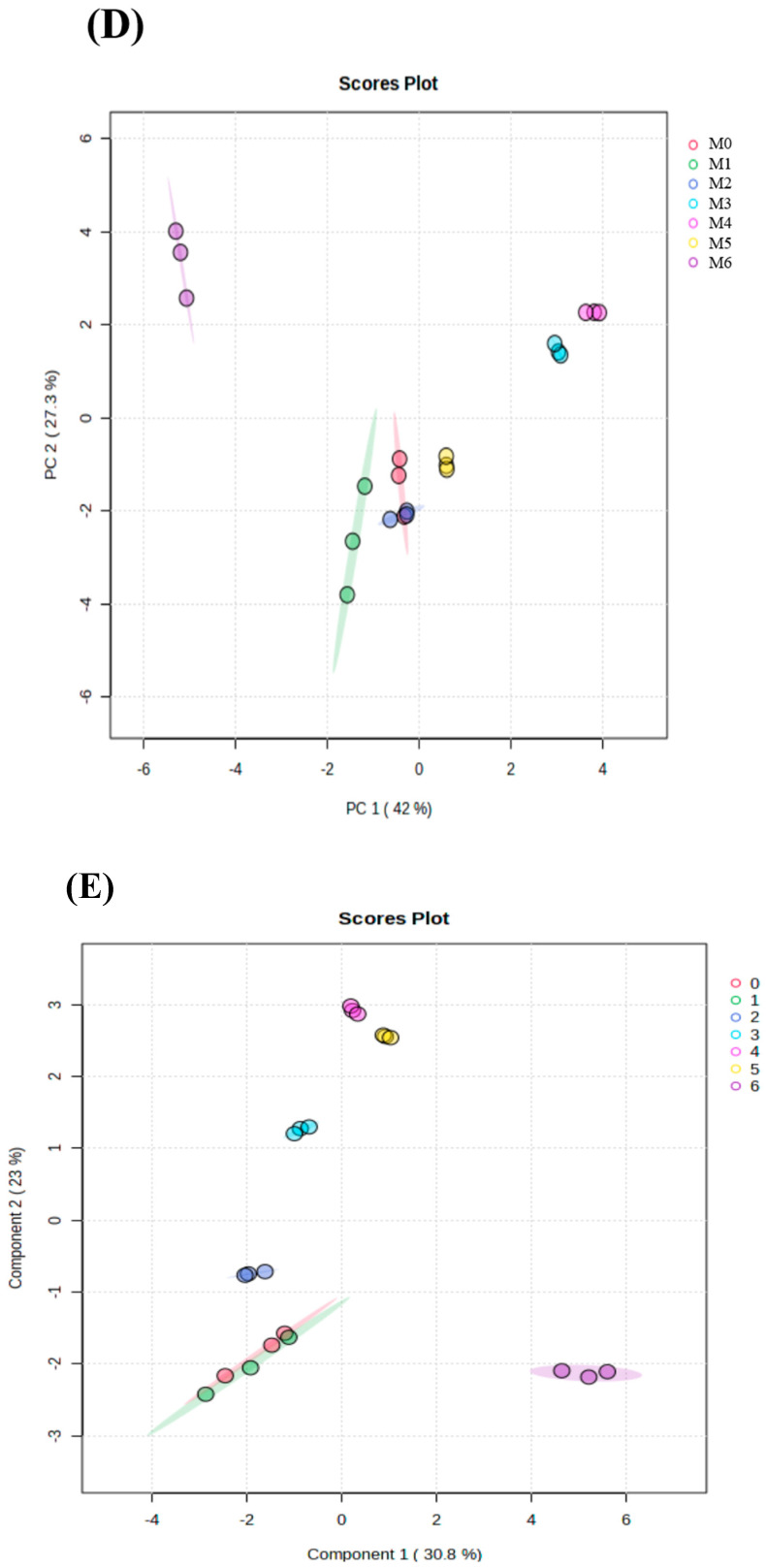

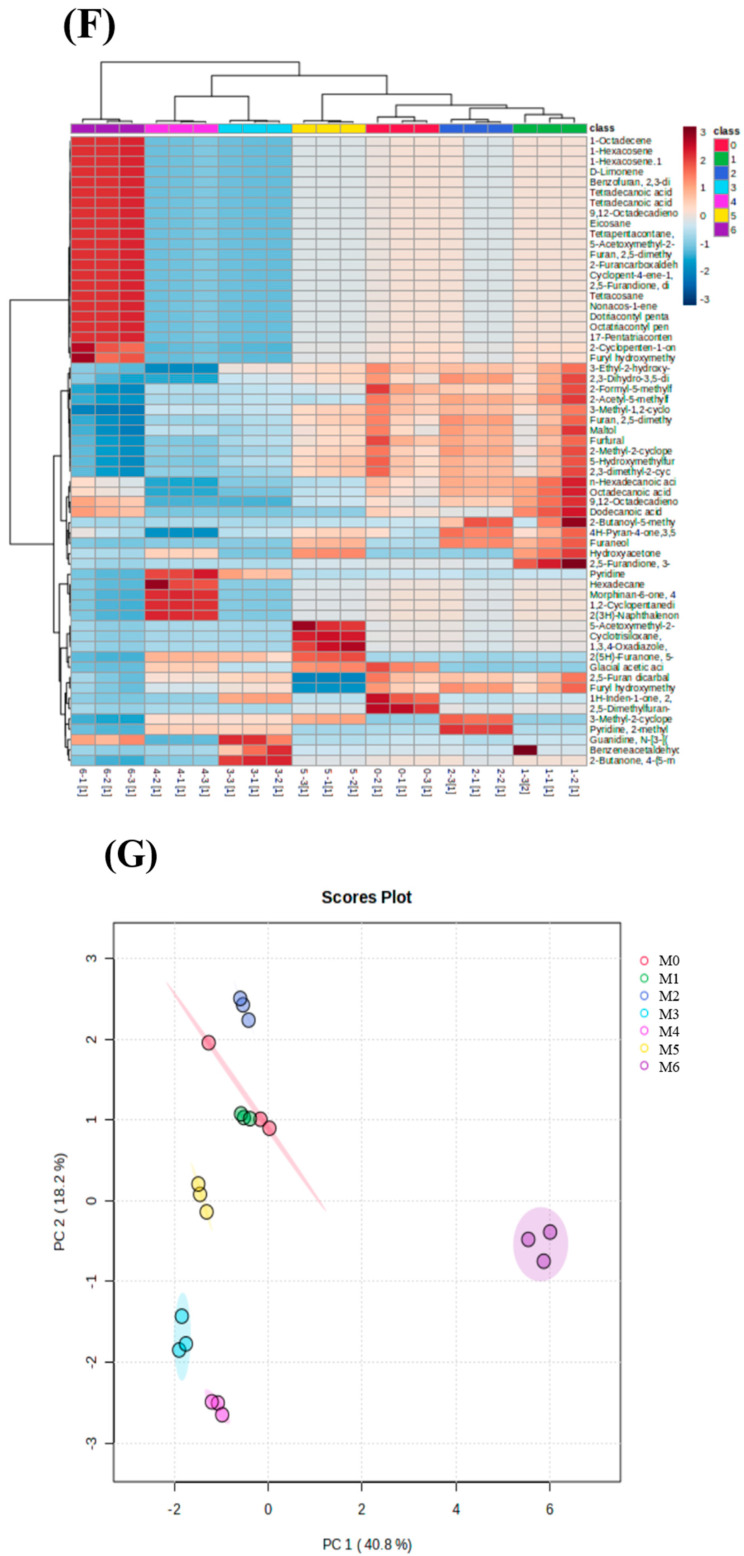

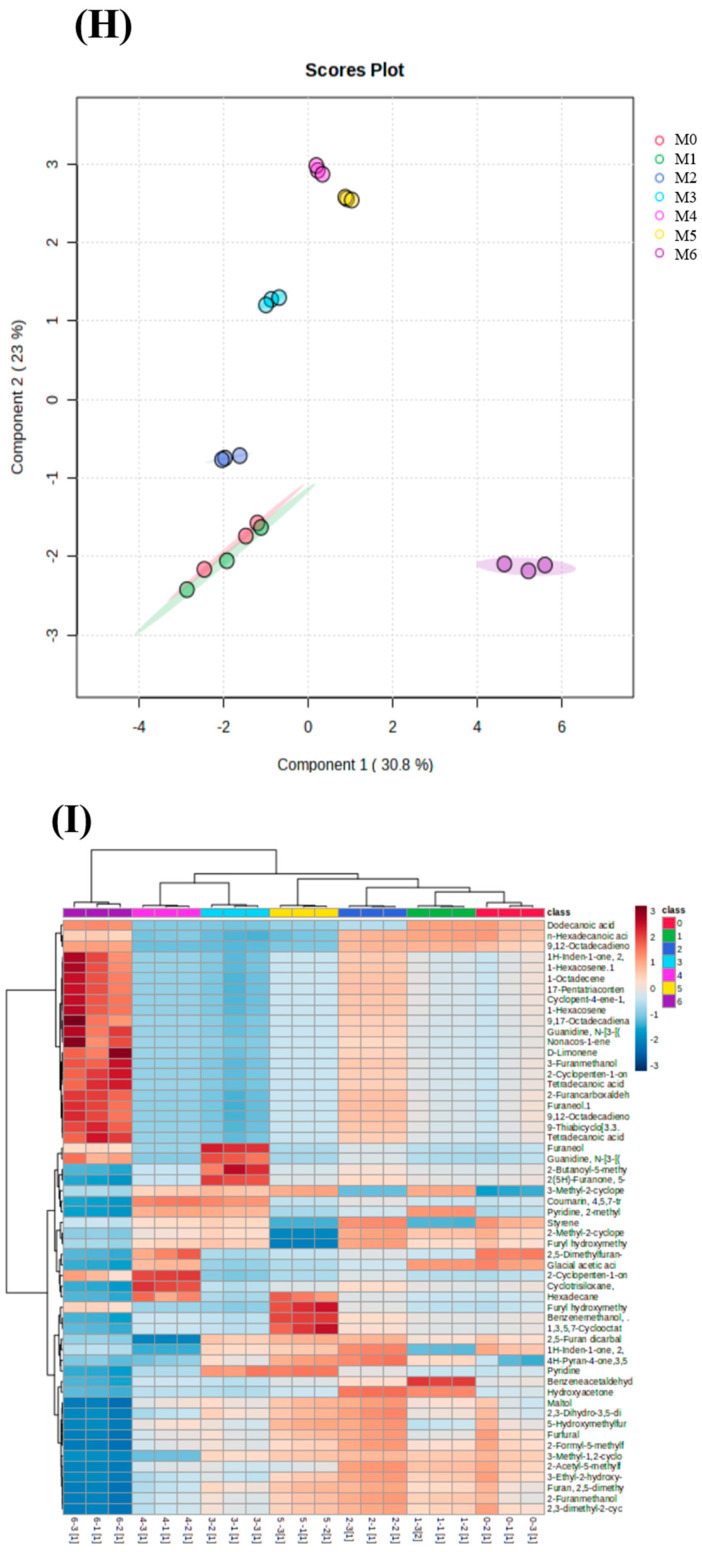
Changes in volatile compounds in melanoidins after pyrolysis. PCA score plot of melanoidins stored at (**A**) 4 °C, (**D**) 20 °C, and (**G**) 35 °C; PLS-DA score plot of melanoidins stored at (**B**) 4 °C, (**E**) 20 °C, and (**H**) 35 °C; heat map for cluster analysis of volatile compounds of melanoidins stored at (**C**) 4 °C, (**F**) 20 °C, and (**I**) 35 °C. Abbreviations: M0, melanoidins that did not be stored from black garlic; M1, melanoidins from black garlic stored for a month; M2, melanoidins from black garlic stored for 2 months; M3, melanoidins from black garlic stored for 3 months; M4, melanoidins from black garlic stored for 4 months; M5, melanoidins from black garlic stored for 5 months; M6, melanoidins from black garlic stored for 6 months.

After the initial analysis, PCA was used for multivariate analysis to further identify differences (Figure 3A,D,G). On PC1 and PC2, MLDs stored under different conditions could be clearly distinguished. The first two principal components accounted for 61.2% and 69.3%. On the positive quadrants of PC1, pyrolysis MLDs stored for 6 months could be effectively separated from MLDs stored for other periods at three storing temperatures. This might be why the contents of heterocyclic compounds and ketones were higher than those in unsored MLDs with a significant difference and why the contents of acids and aliphatic hydrocarbons were lower. On PC2, all contents could be separated at 35 °C, which was perhaps due to each component having significant changes after higher-temperature storage. In summary, most of the components of MLDs stored at 4 °C and 20 °C could be effectively separated, but few differences were noted. Consequently, PLS-DA was applied to investigate further. After treating Py-GC-MS data with PLS-DA, MLD pyrolysis components at different times and temperatures were effectively and clearly distinguished (Figure 3B,E,H). 

Furthermore, the heat map was applied to explore differences among different groups of samples. The components with significant differences among the 4 °C MLD pyrolysis components were 3-methyl-2-cyclopentenone, 2,5-dimethylfuran-3,4 (2H, 5H) -Dione, and 2,5-furan carbaldehyde. Significant differences in the 20 °C MLD pyrolysis components were reflected in 5-hydroxymethylfurfural, 2-formyl-5-methylfuran, and furfural. At 35 °C, the main molecules among MLD components showing significant differences were glacial acetic acid, 3-methyl-1,2-cyclopentanedione, and 2-methyl-2-cyclopentenone. 

Overall, the change in heterocyclic compounds and ketones was the most significant feature during storage. Ketones come from the degradation of sugars. Glacial acetic acid might come from the degradation of saccharides at high temperatures [29] or from ketene after the removal of o-methyl and carbonyl groups [30]. In general, the results show that PCA combined with heat maps and VIP scores could distinguish differences in MLD pyrolysis components at each storage condition. 

### 3.5. The Structural Characteristics of MLDs 

#### 3.5.1. Molecular Weight (MW) Measurement

MLDs are high polymers with different MWs, and the molecules are closely connected. The weight-average MW of MLDs under different storage conditions is shown in Table 2. The MW of MLDs stored at 4 °C decreased from 14,300 Da to 14,175 Da, which means that MLDs were mainly concentrated in the initial stage of the MR with a lower rate of aggregation in the HMW MLD reaction. 

Our results verify that the MW of MLDs increased during storage at 20 °C and 35 °C, which means more HMW MLDs were produced in the middle and late stages. The decrease in MLDs’ MW after storage at 4 °C was due to the massive initial-stage products of the MR, resulting in a decrease in the weight-average MW, which could explain why MLDs stored at 4 °C had higher absorption at 280 nm. The proportion of MLDs with a HMW stored at 20 °C and 35 °C was greater, which is consistent with previous results showing absorption at 420 nm [31]. In brief, higher temperatures accelerated the MR rate and browning degree due to the accelerated formation of HMW MLDs, which is similar to Shiqi Yang’s finding that Chinese liquor MLDs formed in high-temperature distillation had a higher MW [15]. 

#### 3.5.2. FT-IR Analysis 

**Figure 4:**
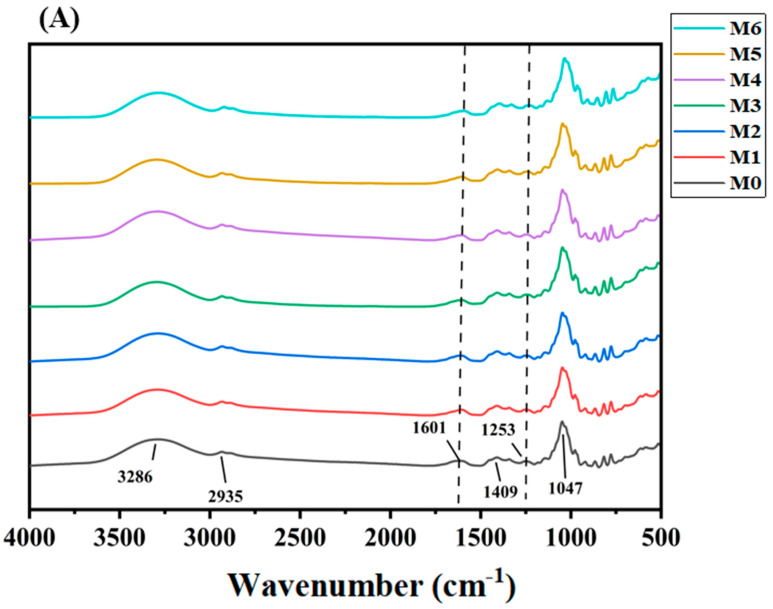

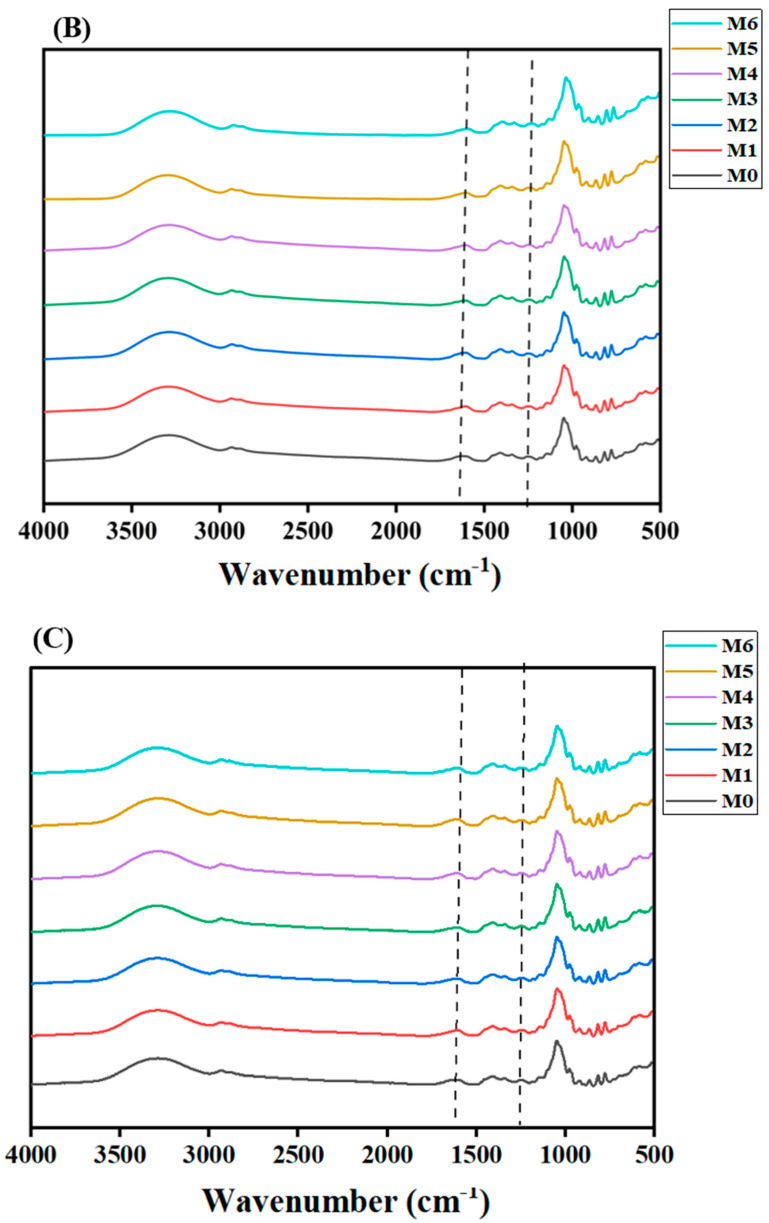
Fourier transform infrared spectroscopy (FT-IR) spectra of melanoidins at (**A**) 4 °C, (**B**) 20 °C, and (**C**) 35 °C. Abbreviations: M0, melanoidins that did not be stored from black garlic; M1, melanoidins from black garlic stored for a month; M2, melanoidins from black garlic stored for 2 months; M3, melanoidins from black garlic stored for 3 months; M4, melanoidins from black garlic stored for 4 months; M5, melanoidins from black garlic stored for 5 months; M6, melanoidins from black garlic stored for 6 months.

Infrared spectroscopy can identify the characteristic groups in the complex molecular structure of MLDs. The spectral range of MLDs was between 4000 cm^−1^ and 450 cm^−1^ (Figure 4). The infrared image of MLDs that were not stored showed six main feature groups: The band at 3286 cm^−1^ was attributed to the -OH stretching mode, indicating the content of hydroxyl groups was high in all MLD samples. The characteristic band at 2935 cm^−1^ was caused by the stretching of CH_2_. The absorption bands at 1601, 1410, 1253, and 1047.43 cm^−1^ were assigned to the C=C, C−H, C−O, and C−O−C groups, respectively. The absorption bands at 1601, 1410, and 1253 cm^−1^ were ascribed to the amide I, II, and III groups, respectively. MLDs under all storage conditions had strong absorption in the above regions, and the peak positions were similar.

The main differences in the infrared spectra were mainly concentrated in the amide I and amide III groups as the storage time and temperature increased, but the difference in storage time was more significant. The MLDs that were not stored peaked at 1622 cm^−1^ (the amide I group), but the peak moved to 1604 cm^−1^ at 4 °C, 1601 cm^−1^ at 20 °C, and 1600 cm^−1^ at 35 °C after 6 months of storage. The MLDs that were not stored peaked at 1254 cm^−1^ (the amide III group), but the peak moved to 1241 cm^−1^ at 4 °C, 1239 cm^−1^ at 20 °C, and 1240 cm^−1^ at 35 °C after 6 months of storage. 

In summary, we identified that the chemical changes in the MR resulted in a change in the infrared spectra of the MLDs due to the alteration of several functional groups. Functional groups (including -NH_2_) may be lost, but they can be increased by the presence of reactions such as Amadori compounds (C=O), Schiff bases (C=N), and pyrazine (C–N) [32]. It has been reported that hydroxyl and amino groups that are consumed during the heating process of brewers’ grains are indicated by the changes in the amide I and amide II bands [33]. These results are similar to those found in a previous study by Yang et al. (2023) on MLDs in distillers’ grains, which showed a change in amide structure that indicated that the MR might change the structure of proteins [15]. Additionally, the change in the functional groups of MLDs during storage was greatly affected by storage time. 

#### 3.5.3. Morphology Analysis

**Figure 5:**
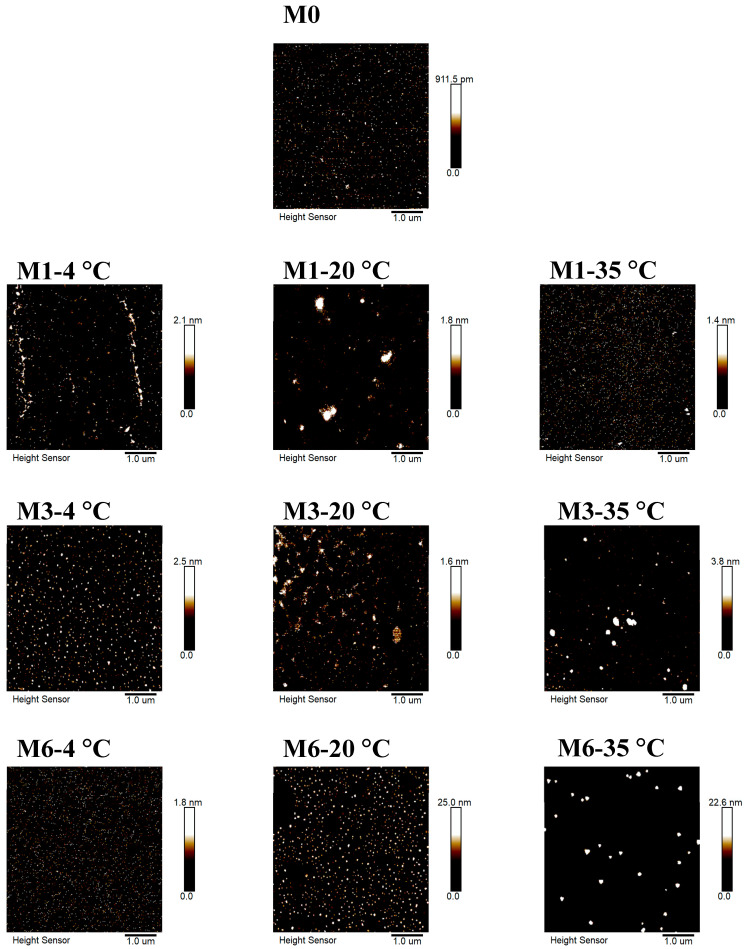
Atomic force microscope images of melanoidins stored at different temperatures. Abbreviations: M0, melanoidins that did not be stored from black garlic; M1, melanoidins from black garlic stored for a month; M3, melanoidins from black garlic stored for 3 months; M6, melanoidins from black garlic stored for 6 months.

The surface morphology of MLDs stored for different periods was obtained via AFM (Figure 5). Surface roughness (Ra), skewness, and kurtosis are parameters commonly applied to estimate the surface morphology of samples measured via AFM and are shown in the Appendix C, Table A3. Ra was applied to assess the surface roughness of samples. The skewness was utilized to assess the symmetry of the sample surface distribution. The peak–valley distribution of the sample surface and the sample height was uniform when the value was 0. The kurtosis was measured according to the convex peaks on the sample surface. The Ra of MLDs stored at 4 °C tended to decrease during storage, especially in the sixth month. In contrast, the Ra tended to increase during storage at 20 °C and 35 °C, particularly in the sixth month. Generally, Ra increased with rising temperature, especially at 20 °C and 35 °C. 

Our results suggest that the changes in surface morphology were consistent with the MW, which could explain the decrease in Ra at 4 °C and the increase in Ra at 20 °C and 35 °C. Low-MW MLDs were more likely to aggregate to form more HMW MLDs [34,35], thus changing the morphology and structure of MLDs [25,36]. The skewness was higher at 35 °C than at other temperatures, which showed that the surface height of the sample was uneven, with large differences. The kurtosis at 35 °C was higher than that at 4 °C and 20 °C, and the convex peaks on the sample surface were more chaotic and irregular [37]. Our previous studies showed that more initial products of the MR, small molecular substances, were generated at 4 °C. The high degree of molecular freedom means it is difficult for MLDs to accumulate at 4 °C. In brief, higher temperatures promoted the production of macromolecular substances in the middle and late stages of the MR, and macromolecules intertwined with each other to increase the roughness and the degree of irregularity. This might be due to the fact that the hydrogen bonds and ionic and hydrophobic interactions directly related to the AFM structure were affected by temperature [10]. However, there is limited research on the mechanism of temperature-induced molecular surface morphology change, and this topic needs to be investigated.

### 3.6. Antioxidant Activity 

**Figure 6:**
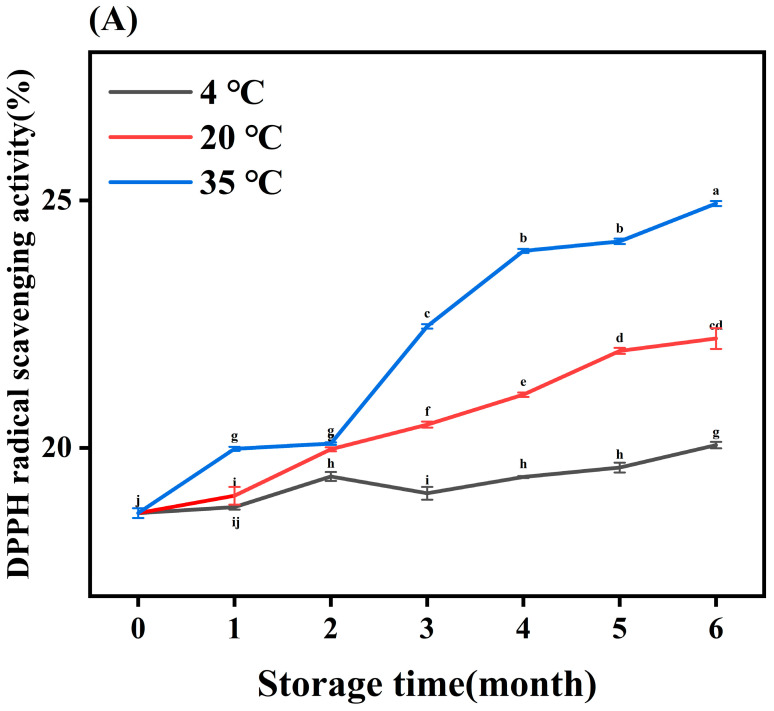

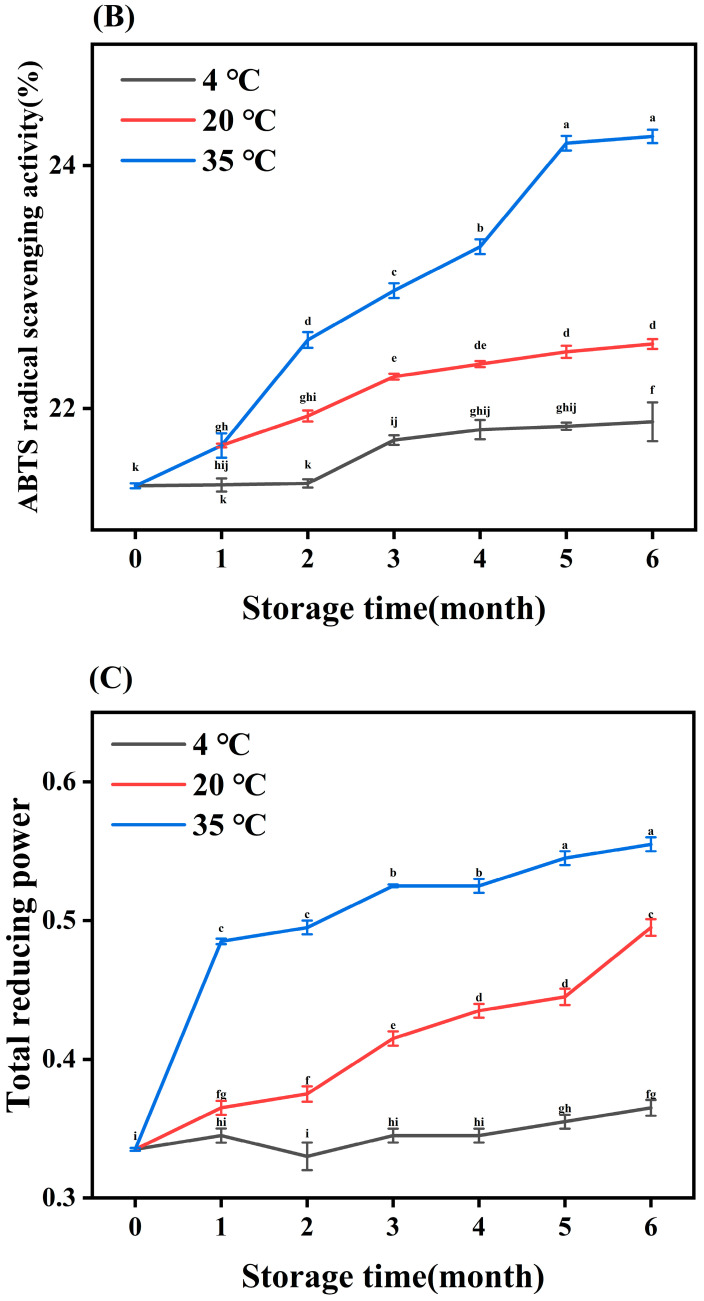
Antioxidant activity of melanoidins. DPPH, ABTS, and total reducing power of melanoidins stored at (**A**) 4 °C, (**B**) 20 °C, and (**C**) 35 °C. Different letters (a–k) within the same absorbance are significantly different at *p* < 0.05, as analyzed using Duncan’s multiple range test.

The DPPH, ABTS radical scavenging activities, and total reducing power were evaluated based on the antioxidant properties of natural botanicals. MLDs under different storage conditions possessed excellent antioxidant activity (as shown in Figure 6), and these results agree with those of the study by Wu et al. (2020) [35], which indicated MLDs play a major role in BG’s antioxidant activity. As shown in Figure 6A, the DPPH radical scavenging activity of MLDs stored for 6 months increased by 33.96% for those stored at 35 °C, 18.41% at 20 °C, and 3.82% at 35 °C compared with the control group, and the scavenging activity increased constantly with the storage temperature. It was found by Liu et al. (2016) [38] that the DPPH radical scavenging activity of vinegar MLDs increased by 63% after heat treatment. The ABTS free radical scavenging ability and total reducing power of BG were improved significantly with an increase in temperature (shown in Figure 6B,C). This is similar to the previous report that the ABTS radical scavenging activity of MLDs formed from bread crust increased with roasting time [39]. Yan et al. (2011) [40] also reported that the reducing power of Maillard reaction products from the psicose–lysine model reaction was enhanced with prolonged heating, and the main factor was MLDs. 

It could be confirmed that the antioxidant activity increased with storage temperature, and there was a positive linear correlation between browning and antioxidant activity. Several studies reported that an increase in HMW components in MLDs during hot processing might increase the antioxidant capacity of the components [41], which is in accordance with the above MW measurement results. In addition, the antioxidant properties of MLDs have been attributed to their capacity to chelate transition metals and hydrogen donor tendency [42], and the increased storage temperature may have enhanced these abilities. Additionally, the antioxidant activity of MLDs is associated with phenolic compounds, which is consistent with the results of the total phenolic assay. These results show that there was a significant difference in antioxidant activity at 4 °C but no significant difference in the MLD content. In brief, the antioxidant capacity of MLDs was positively correlated with storage time, which was related to browning degree, MW, metal-ion chelating activity, hydrogen donor tendency, and binding compounds. The results show that BG MLDs have great potential as food additives and antioxidants.

## 4. Conclusions

In summary, this study reveals the differences in the chemical composition, structure, and antioxidant activity of MLDs stored for different times and at different temperatures. BG underwent a continuous MR during the storage process, resulting in the formation of MLDs. The MW of the MLDs also increased with storage time and temperature. Free amino acids were continuously integrated into the MLD framework. The increase in nitrogen content also suggested an increase in the amino acid content in the MLD structure during its formation. The MR generated more products at a temperature of 4 °C during the initial stage, while higher temperatures (>20 °C) tended to promote the formation of HMW compounds. Ketones and heterocycles were derived from precursor substances, which played significant roles in MLD formation. Therefore, the smoothest MLD surfaces were observed at 4 °C, while rougher MLD surfaces were formed at higher temperatures. In addition, the antioxidant capacity was positively correlated with storage temperatures. In summary, temperature had an impact on the composition, structure, and antioxidant activity of MLDs. Further research is needed to investigate the potential changes in their functional characteristics and food safety as a result.

## Figures and Tables

**Table 1:** Elemental compositions of melanoidins stored at 4 °C, 20 °C, and 35 °C.

Temperature	Storage Time (Month)	O (%)	C (%)	H (%)	N (%)
4 °C	0	55.81 ± 0.02 ^a^	36.07 ± 0.49 ^l^	6.8 ± 0.01 ^f^	1.03 ± 0.01 ^g^
	1	54.07 ± 0.01 ^c^	38.01 ± 0.06 ^j^	6.64 ± 0.01 ^i^	1.09 ± 0.01 ^f^
	2	52.76 ± 0.02 ^ef^	38.76 ± 0.07 ^f^	6.86 ± 0.02 ^e^	1.12 ± 0.01 ^e^
	3	53.41 ± 0.06 ^d^	38.45 ± 0.06 ^g^	6.74 ± 0.01 ^h^	1.11 ± 0.01 ^e^
	4	52.41 ± 0.02 ^fg^	38.99 ± 0.01 ^e^	6.99 ± 0.05 ^c^	1.11 ± 0.02 ^e^
	5	52.58 ± 0.03 ^fg^	39.07 ± 0.01 ^cd^	7.00 ± 0.01 ^c^	1.12 ± 0.01 ^e^
	6	52.59 ± 0.04 ^fg^	39.03 ± 0.01 ^d^	7.01 ± 0.01 ^c^	1.12 ± 0.01 ^e^
20 °C	0	55.81 ± 0.02 ^a^	36.07 ± 0.01 ^l^	6.80 ± 0.02 ^f^	1.03 ± 0.01 ^g^
	1	54.74 ± 0.04 ^b^	36.35 ± 0.16 ^k^	6.77 ± 0.01 ^g^	1.02 ± 0.01 ^g^
	2	53.17 ± 0.07 ^d^	38.17 ± 0.07 ^i^	6.92 ± 0.02 ^d^	1.02 ± 0.01 ^g^
	3	52.61 ± 0.02 ^fg^	38.38 ± 0.53 ^gh^	6.86 ± 0.01 ^e^	1.11 ± 0.02 ^e^
	4	52.00 ± 0.03 ^h^	38.37 ± 0.03 ^h^	6.99 ± 0.05 ^c^	1.15 ± 0.02 ^cd^
	5	52.75 ± 0.01 ^d^	39.05 ± 0.03 ^cd^	6.95 ± 0.01 ^d^	1.13 ± 0.01 ^e^
	6	52.59 ± 0.02 ^fg^	39.07 ± 0.02 ^cd^	7.00 ± 0.02 ^c^	1.13 ± 0.01 ^e^
35 °C	0	55.81 ± 0.02 ^a^	36.07 ± 0.09 ^l^	6.80 ± 0.01 ^f^	1.03 ± 0.01 ^g^
	1	53.58 ± 0.04 ^d^	38.21 ± 0.03 ^i^	6.77 ± 0.04 ^fg^	1.13 ± 0.01 ^e^
	2	53.16 ± 0.05 ^de^	38.40 ± 0.01 ^gh^	7.06 ± 0.01 ^b^	1.15 ± 0.01 ^cd^
	3	52.50 ± 0.05 ^fg^	39.11 ± 0.11 ^bc^	6.99 ± 0.03 ^c^	1.15 ± 0.01 ^cd^
	4	52.19 ± 0.06 ^gh^	39.19 ± 0.05 ^a^	7.04 ± 0.01 ^b^	1.19 ± 0.01 ^a^
	5	52.51 ± 0.03 ^fg^	39.03 ± 0.02 ^cd^	7.11 ± 0.01 ^a^	1.17 ± 0.01 ^b^
	6	52.32 ± 0.05 ^fgh^	39.17 ± 0.01 ^ab^	7.12 ± 0.01 ^a^	1.18 ± 0.01 ^ab^

Data are presented as mean ± SD (n = 3). Data with different superscript letters (a–l) in the same row are significantly different (*p* ≤ 0.05).

**Table 2:** Weight-average molecular weight (MW) of high-molecular-weight melanoidins (Da) stored at 4 °C, 20 °C, and 35 °C.

Storage Time (Month)	Temperature		
	4 °C	20 °C	35 °C
0	14,300 ± 203 ^ab^	14,300 ± 203 ^ab^	14,300 ± 203 ^ab^
1	14,295 ± 124 ^ab^	14,310 ± 148 ^ab^	14,326 ± 117 ^ab^
2	14,196 ± 147 ^b^	14,309 ± 104 ^ab^	14,320 ± 139 ^ab^
3	14,203 ± 118 ^b^	14,305 ± 150 ^ab^	14,342 ± 112 ^ab^
4	14,181 ± 132 ^b^	14,326 ± 123 ^ab^	14,341 ± 171 ^ab^
5	14,171 ± 115 ^b^	14,320 ± 112 ^ab^	14,456 ± 121 ^a^
6	14,175 ± 112 ^b^	14,325 ± 102 ^ab^	14,449 ± 121 ^a^

Data are presented as mean ± SD (n = 3). Data with different superscript letters (a,b) in the table are significantly different (*p* ≤ 0.05).

## Data Availability

The data presented in this study are available on request from the corresponding author.

## Appendix A

**Table A1:** Content of amino acids.

Temperature	Storage Time (Month)	Arg (mg·100g^−1^)	P-Ser (mg·100g^−1^)	Glu (mg·100g^−1^)	Lys (mg·100g^−1^)	Asp (mg·100g^−1^)	Ser (mg·100g^−1^)	Thr (mg·100g^−1^)	Met (mg·100g^−1^)	His (mg·100g^−1^)
4 °C	0	844.26	119.12	65.98	51.35	44.79	31.50	27.95	23.90	4.53
	3	850.63	67.91	72.42	55.40	41.78	31.11	27.49	26.81	4.29
	6	673.82	39.34	52.45	39.76	22.75	22.13	17.11	15.69	2.04
20 °C	0	844.26	119.12	65.98	51.35	44.79	31.50	27.95	23.90	4.53
	3	1016.12	75.99	89.09	65.23	53.46	39.60	32.96	29.00	6.12
	6	737.95	33.19	53.90	47.19	19.11	23.25	15.67	16.53	3.58
35 °C	0	844.26	119.12	65.98	51.35	44.79	31.50	27.95	23.90	4.53
	3	758.45	68.66	67.88	38.15	42.95	32.35	31.34	26.50	3.94
	6	798.00	30.26	55.61	48.44	17.98	20.68	15.50	16.14	3.51

## Appendix B

**Table A2:** Pyrolysis–gas chromatography–mass analysis of melanoidins.

Temperature	Storage Time (Month)	Heterocyclic Compounds		Acids		Aliphatic Hydrocarbons		Ketones		Alcohols		Aromatic Hydrocarbons		Other Compounds	
		n	P (%)	n	P (%)	n	P (%)	n	P (%)	n	P (%)	n	P (%)	n	P (%)
4 °C	0	22	56.6	5	19.68	21	9.28	22	5.93	5	1.79	8	4.39	18	2.33
	1	24	57.11	4	16.11	17	9.01	23	6.37	5	2.27	7	4.86	23	4.27
	2	29	55.74	5	11.09	26	5.62	24	6.19	4	2.7	7	4.5	15	14.16
	3	19	61.29	9	4.53	15	19.38	25	5.55	7	2.14	5	3.36	13	3.75
	4	19	59.01	12	16.44	17	5.15	17	5.89	6	2.33	4	2.31	14	8.86
	5	23	60.79	16	19.16	19	3.09	25	5.97	7	2.69	3	2.61	12	5.69
	6	23	59.2	16	20.12	19	1.83	15	6	4	2.78	3	2.63	15	7.44
	Total number	101		103											
20 °C	0	22	56.6	5	19.68	21	9.28	22	5.93	5	1.79	8	4.39	18	2.33
	1	29	61.9	6	17.54	19	5.76	18	5.96	7	2.31	3	5.02	24	1.51
	2	33	66.97	7	12.91	23	5.71	16	5.47	8	2.94	6	5.36	14	0.64
	3	23	69.46	11	13.66	19	1.12	30	7.43	6	2.21	6	4.56	17	1.56
	4	27	69.52	15	12.43	16	4.24	24	7.02	6	2.24	5	3.09	13	1.47
	5	28	73.22	10	11.63	16	2.72	29	8.59	5	2.41	3	1.41	11	0.01
	6	28	72.5	9	11.78	22	1.76	18	8.9	7	2.65	3	2.24	13	0.17
	Total number	190		63		136		157		44		34		110	
35 °C	0	22	56.6	5	19.68	21	9.28	22	5.93	5	1.79	8	4.39	18	2.33
	1	31	62.1	7	18.91	21	5.73	27	6.05	6	2.31	5	3.4	19	1.5
	2	24	69.14	8	11.89	25	4.78	26	5.95	9	2.26	9	5.23	17	0.75
	3	29	73.57	10	2.77	24	6.82	29	7.8	9	2.39	7	4.29	20	2.36
	4	29	70.75	12	13.12	18	2.5	28	8.65	5	1.89	4	2.87	17	0.22
	5	25	79.84	5	1.94	21	3.91	17	8.67	5	2.53	4	2.21	15	0.9
	6	32	78.89	6	4.94	20	1.69	20	9.2	5	2.69	3	2.3	15	0.29
	Total number	192		53		150		169		44		40		121	

n, the number of compounds species. P, the proportion of compounds.

## Appendix C

**Table A3:** Atomic force microscope surface topography indices.

Temperature	Storage Time (Month)	Mean Roughness (R_a_, nm)	Skewness	Kurtosis
4 °C	0	0.147	2.29	56
	1	0.248	2.44	14.1
	3	0.332	1.9	8.18
	6	0.259	1.85	9.44
20 °C	0	0.147	2.29	56
	1	0.282	4.14	50.8
	3	0.269	1.41	8.64
	6	2.830	2.8	10.9
35 °C	0	0.147	2.29	56
	1	0.198	12.2	424
	3	0.588	13.6	276
	6	1.070	10.3	124
